# Editorial: Women in CNS drug delivery: 2022–2024

**DOI:** 10.3389/fddev.2024.1400937

**Published:** 2024-10-15

**Authors:** Szilvia Veszelka, Malgorzata Burek, Sarah Ann Thomas

**Affiliations:** ^1^ HUN-REN Biological Research Centre, Szeged, Hungary; ^2^ University Hospital Würzburg, Department of Anaesthesiology, Intensive Care, Emergency and Pain Medicine, Würzburg, Germany; ^3^ King’s College London, Institute of Pharmaceutical Science, London, United Kingdom

**Keywords:** women in science, drug delivery, gender gap, CNS disease, blood-brain barrer

At present, less than 30% of researchers worldwide are women. The underrepresentation of women in scientific research is influenced by several factors, including gender discrimination, societal conditions, and historical numerical imbalances (Astegiano et al., 2019; Avolio et al., 2020; Sá et al., 2023). The consequences of this underrepresentation are significant and include a gender productivity gap, and gender bias in several research fields (Astegiano et al., 2019; Avolio et al., 2020). The underrepresentation of women in scientific research also affects the diversity of research outputs, as women’s contributions are often not recognised and are fragmented, further compounding the gender gap in scientific outputs (Ross et al., 2022). New initiatives and interventions are needed to change the underrepresentation and unacknowledged role of women in scientific research, with efforts to improve and encourage women’s participation in research and scientific work, including promoting collaboration between women scientists. Women in academic fields face many barriers to promotion and career advancement, including gender bias in the evaluation of research results, individualism, glass ceilings and difficulties in reconciling work and family life (Avolio et al., 2020). However, science and gender equality are essential to ensure sustainable development, as emphasised by UNESCO. In order to change traditional mindsets, gender equality must be promoted and the unacknowledged and undervalued role of women in scientific research be carefully considered to select key success descriptors for individuals and groups for future development and dissemination. In this way, stereotypes could be overcome and girls and women would be supported and encouraged to choose science, technology, engineering and mathematics (STEM) careers.

This Research Topic offered a platform to promote the recent work of women scientists. The works presented in this article Research Topic highlight the diversity of research conducted across the breadth of CNS drug delivery research, and presents studies focused on blood-brain barrier (BBB) penetration of eflornithine, a neuropharmacon against Human African Trypanosomiases (HAT), high-throughput screening (HTS) to identify drugs that increase BBB permeability, the contribution of glioblastoma vasculature to malignancy, and the role of microRNAs in the regulation of the BBB in ischemic stroke.

Pharmaceutical treatment of disorders of the central nervous system (CNS), like stroke, brain tumors, or neuroinflammation caused by parasites, is difficult because the BBB restricts the penetration of drugs to the brain. The anatomical basis of the BBB is formed by brain endothelial cells lining the cerebral microvasculature. The main roles of the BBB are the creation of ionic homeostasis for neuronal functions, the supply of CNS with nutrients and the protection of neural cells from toxic insults. Brain endothelial cells control the traffic of cells and molecules from blood to brain by mechanisms including interendothelial tight junctions (TJ), which block the paracellular passage of hydrophilic drugs, specific transport pathways, active efflux transporters and enzymes which remove or metabolize xenobiotics and drugs.

There is a huge research effort to increase drug delivery to the CNS. To reach this goal better understanding of the BBB functions and especially its transport systems, and their use for targeted drug delivery is indispensable (Neuwelt et al., 2011). Due to the diversity of neurotherapeutics and the complexity of the BBB functions and regulation of transport systems, several strategies and new drug delivery and targeting systems are currently being examined which show great potential future medical application.

The clearer understanding of the transport pathways of the BBB and the potential transporters of possible neuropharmacons is required to design more effective drugs and treatment approaches. Eflornithine is a clinically used second-line treatment for HAT gambiense at stage 2 associated with parasite entry into the brain resulting in a variety of neurological disturbances. Research by Watson et al. at King’s College London revealed that eflornithine is a substrate for transporters expressed in brain endothelial cells - in particular the cationic amino acid transporter, system y^+^, and the organic cation transporters. These transporters mediate eflornithine delivery across the BBB in sufficient concentrations to treat meningo-encephalitic stage gambiense HAT. These results proved that the cationic amino acid transporters may be exploited by drugs to cross the BBB and has potential as a target for drug candidates to enter into the brain.

In the treatment of neurological diseases, including brain tumors, strategies to increase the penetration of therapeutic drugs in efficient amount across the BBB are urgently needed. One possible approach to achieve this is to temporarily increase BBB permeability using small molecules. Curtaz et al. present a new approach to measure paracellular permeability without using transwell inserts. They used 96-well plates coated with biotin-conjugated gelatin to seed human CD34+-derived brain-like endothelial cells, which were then exposed to 1,287 compounds. NeutrAvidin TM was added at the end of treatment and bound to biotin-conjugated gelatin in wells with increased paracellular permeability and its signals were detected with a microplate reader. Validation of results in the transwell system for selected compounds confirmed the results obtained in 96-well plates. With this method, 232 substances were identified that cause at least a 50 percent increase in BBB permeability and can therefore potentially be used in clinical practice to increase CNS drug delivery of systemically administered drugs. A complete list of small molecules affecting paracellular BBB permeability can be found here: https://pubchem.ncbi.nlm.nih.gov/bioassay/1963498.

One of the most challenging CNS diseases are brain tumours, particularly glioblastomas. Various preclinical research tools and techniques, including *in vitro* and *in vivo* models, have been developed to mimic the characteristics and behaviour of human glioblastoma, thereby bridging the gap between preclinical and clinical studies. A review by Pacheco et al. evaluates the importance of blood vessels in glioblastoma and the advances in 3D vascularised glioblastoma models. In recent years, innovations in microscale tissue engineering technologies have enabled significant breakthroughs in 3D tumour modelling, particularly in the development of vascular tumour models. The new 3D models can contain different cell populations and mimic native tumour characteristics, such as oxygen/nutrient gradients and tumour cell communication with the neighbouring microenvironment.

Sun et al. highlighted the critical role of micro RNAs as significant biomarkers of CNS injuries and as important therapeutic targets of the future. Under physiological and pathological conditions, the function of miRNAs is one of the key regulators of the BBB. Altered miRNAs levels have been observed in cerebral ischemia, reperfusion injury, and dysfunction of the BBB. MiRNAs regulate BBB integrity by modulating gene expression by targeting mRNA transcripts. Several mRNA transcripts at the BBB are direct miRNA targets e.g., occludin, Zonulae occludentes (ZO), Junctional adhesion molecule (JAM), claudin-1, P-glycoprotein (Pgp) or Glucose transporter (Glut1). The mechanisms of action of miRNA-based therapeutic approach are based on anti-inflammatory activities such as inhibition of astrocyte activation, cytokine secretion, and leukocyte extravasation (Salvador et al., 2015). However, challenges remain in fully understanding the complex molecular interactions involved in BBB regulation. MiRNAs are potential therapeutic targets for preventing BBB dysfunction and maintaining its integrity in neurological disorders (Harati et al., 2022).

In conclusion, the BBB presents a significant challenge in the treatment of brain diseases, and innovative drug delivery strategies targeting the BBB are crucial for overcoming this obstacle and improving therapeutic outcomes. This Research Topic of articles highlights recent contributions of women scientists and their groups to this research endeavour.
